# Information Seeking and Processing during the Outbreak of COVID-19 in Taiwan: Examining the Effects of Emotions and Informational Subjective Norms

**DOI:** 10.3390/ijerph19159532

**Published:** 2022-08-03

**Authors:** Shu-Chu Sarrina Li, Shih-Yu Lo, Tai-Yee Wu, Te-Lin Chen

**Affiliations:** 1 Institute of Communication Studies, National Yang Ming Chiao Tung University, Hsinchu 30010, Taiwan; shihyulo@nycu.edu.tw (S.-Y.L.); taiyeewu@nycu.edu.tw (T.-Y.W.); 2 Institute of Applied Arts, National Yang Ming Chiao Tung University, Hsinchu 30010, Taiwan; inspire.hs09@nycu.edu.tw

**Keywords:** information seeking and processing, information-processing modes, COVID-19, RISP, informational subjective norms

## Abstract

Adopting the model of risk information seeking and processing (RISP) as a theoretical framework, the objective of this study was to investigate the factors that prompted individuals’ information-seeking and -processing behaviors during the COVID-19 pandemic in Taiwan. There were two unique aspects in this study: one was to adopt specific emotions to investigate the impact of negative emotions, and the other was to examine the effect of informational subjective norms (ISNs) on information-seeking and -processing behavior. An online survey was conducted by a professional polling company, and a stratified random sampling method was employed, using gender, age, education, personal income, and residential areas as strata to select participants. This study obtained 1100 valid questionnaires. The results showed that (1) risk perception did not exert any significant impacts on respondents’ perceived information insufficiency; (2) risk perception exerted a powerful impact on respondents’ ISNs, which, in turn, positively affected their information insufficiency; (3) the respondents who experienced fear were found to have a high probability of using a systematic-processing mode, while the respondents who experienced anger were more likely to adopt a heuristic-processing mode to process information; and (4) the use of a systematic-processing mode was positively associated, while the use of a heuristic-processing mode was negatively associated, with information-seeking behavior.

## 1. Introduction

In early March 2020, COVID-19 was declared a global pandemic by the WHO. This disease differs greatly from past health crises because it has disrupted the lives of most people across the world for more than two years, thus severely impacting the world economy [1,2]. Thus far, the rate of the COVID-19 contagion has been exceptionally rapid, with multiple variants continually emerging. Because people are easily infected by COVID-19 via personal contact, many cities have been forced to pause most of their regular operations to mitigate the transmission of this disease. While several types of internationally certified vaccines are available, medical experts are unsure whether these vaccines will enable people to protect themselves against the variants of COVID-19 [2,3].

During the outbreak of COVID-19, people in Taiwan have faced high uncertainty. Past studies have shown that, when individuals experience a high degree of uncertainty, they seek information to reduce their uncertainty [2]. Adopting the model of risk information seeking and processing (RISP) as a theoretical framework, the objective of this study was to examine the factors that affected individuals’ information seeking and processing during the outbreak of COVID-19 [4,5]. During the outbreak of contagious diseases such as COVID-19, releasing timely and accurate information to the public is a vital step in managing the health crisis; thus, the results of this study provide governments and health practitioners with a good understanding of what types of information need to be offered and what factors influence people’s information-seeking and -processing behaviors during health crises.

The RISP model identifies several cognitive, psychosocial, and communicative factors that affect when people seek risk information and how they process this information [5,6]. Several assumptions are proposed in the RISP model: (1) individuals’ risk perception and affective response activate individuals’ perceived information insufficiency, that is, individuals’ need for more information; (2) individuals are predicted to adopt either a thoughtful or superficial way to process information depending on their perceived degrees of information insufficiency, which, in turn, influences their attitudes and behaviors toward the risk [4,5,7]; and (3) affective response is derived from risk perception, which is mostly based on negative emotions such as fear, worry, and anger.

In regard to affective response, as indicated by the meta-analysis conducted by Yang et al. (2014), except worry, all 13 studies they reviewed adopted multiple emotions to measure affective response [4]. That is, these studies added several specific emotions together as affective responses to examine their impacts on information-seeking and -processing behavior. According to the appraisal-tendency framework (ATF), fear and anger are classified as negative emotions, but the two emotions lead individuals to attend to different aspects of content, thus yielding markedly different judgments and decision-making [8,9,10]. That is, the approach of adding several specific emotions together as affective responses does not allow scholars to identify the effect of each emotion on information-seeking and -processing behaviors. Furthermore, Yang et al. (2014) indicated that the impact of affective response might have been underestimated because the majority of the studies in their meta-analysis grouped several specific emotions to represent an affective response [7].

Two recent studies [6,7] examined the effects of discrete emotions on individuals’ information seeking and processing. Yang et al.’s study (2019) found that respondents’ political ideology, rather than discrete emotions, played a significant role in determining their information processing behaviors [7]. Another study by Lu et al. (2021) investigated the issue of COVID-19, but their study did not examine the relationships between discrete emotions and information processing behaviors [6]. Few studies in the literature have examined the relationship between discrete emotions and individuals’ information-seeking behaviors. Therefore, the uniqueness of this study was to adopt discrete emotions instead of summing up several discrete emotions to investigate the impact of negative emotions on information-seeking and -processing behavior.

Another unique aspect of this study was the important role played by ISNs, which constitute the social expectation that people in a society should obtain sufficient information to help them deal with a given risk [4]. According to the RISP model, ISNs are mainly affected by individuals’ risk perception, which subsequently activates their perception of information insufficiency [4,5]. Taiwanese people belong to a collectivist culture that places a high priority on the interests of society rather than on individuals’ interests [1]. Therefore, during the outbreak of COVID-19, the government in Taiwan executed several strict orders, such as wearing face masks in public and avoiding mass gatherings, to control the spread of the disease. Most people not only carefully followed these orders but also helped supervise other people in abiding by these rules [9,11], a situation that differed greatly from those in other countries. For example, during the outbreak of the pandemic, several mass protests were mobilized in the US and Europe against the order that required people to wear face masks in public, and these protesters deemed this order a severe violation of personal freedom [12,13,14]. This study expects that ISNs play a more important role than other variables of the RISP model in people’s information seeking and processing during the outbreak of COVID-19.

## 2. Literature Review and Hypotheses

The RISP model was originally developed based on two theoretical models—the theory of planned behavior (TPB) [15] and the heuristic-systematic model (HSM) [16,17]. The RISP model identifies the factors that are associated with two information behaviors—information seeking and processing. Among several factors, information insufficiency is the mechanism that drives individuals to seek out and process information. Information insufficiency originates from the HSM’s sufficiency principle, which assumes that individuals will make all possible efforts to acquire an adequate degree of confidence that allows them to satisfactorily achieve their processing objectives [4]. According to the RISP model, risk characteristics such as severity and susceptibility affect individuals’ risk perception, which, in turn, influences their affective response and information insufficiency. Information seeking is prompted by an individual’s perceived information insufficiency, which is defined as the perceived gap between the individual’s existing knowledge and his or her sufficiency threshold. Information processing, which can be divided into a more thoughtful information process and a more superficial information process, is defined as the way individuals handle the acquired information. A more thoughtful information process involves systematic processing that relies more on cognitive resources and uses an analytic and in-depth manner to process information, whereas a more superficial information process relies less on cognitive resources but uses a shortcut to process information [4,18]. The RISP model has been applied to examine a variety of risk issues, including obesity [19], flooding [20], air pollution [21], Zika virus [22], and COVID-19 [1,23].

### 2.1. Risk Perception, Emotions, and Information Seeking and Processing

When Giffin, Dunwoody, and Neuwirth (1999) proposed RISP, they regarded the process of seeking and processing information as containing two stages—cognition and emotion—both of which trigger individuals’ need to seek and process relevant information [5]. Risk perception is the foremost factor in the cognition stage, while affective response, including fear, worry, and other emotions, occurs in the emotion stage. Both result in individuals’ information-seeking behavior [23].

One key variable in the RISP model—risk perception—is usually affected by individuals’ perceived characteristics of the risk, such as its severity and susceptibility. When individuals perceive a risk as being more severe and susceptible, they tend to produce more negative emotions toward this risk, prompting them to have a higher level of perceived information insufficiency. According to the RISP model, the severity and susceptibility of a given risk exert a direct effect on individuals’ information insufficiency, which subsequently urges them to seek and process relevant information. Risk characteristics also elicit negative emotions, such as fear or anxiety, which lead individuals to perceive that the relevant risk information is insufficient. Thus, risk characteristics exert indirect impacts on individuals’ perceived information insufficiency through negative emotions [4,18].

During the outbreak of COVID-19, cities in Taiwan were under lockdown several times when confirmed case numbers were high. To date, strict border control has been in place, and most people in Taiwan have not been out of the region for more than two years. Therefore, most people in Taiwan regard COVID-19 as a highly threatening disease. Based on the propositions of RISP, this study developed the following hypotheses:

**H1:**  *Respondents’ perceived severity is positively associated with their perceived COVID-19 information insufficiency.*

**H2:**  *Respondents’ perceived susceptibility is positively associated with their perceived COVID-19 information insufficiency.*

According to the appraisal-tendency framework (ATF), discrete emotions lead individuals to adopt specific cognitive and motivational processes, which subsequently influence what content they attend to and the depth of their thought. As a consequence, different emotions result in different judgments and decisions. Past studies [24,25] have tended to take a valence approach that classifies emotions into positive and negative emotions. That is, scholars tended to classify emotions into positive and negative emotions. Recently, scholars [26,27] have suggested using discrete emotions instead of positive/negative emotions. Several studies showed that even emotions that are classified as negative emotions, such as fear and anger, lead individuals to attend to different aspects of content, thus yielding markedly different judgments and decision-making. However, recent studies [26,27] have demonstrated that emotions sharing the same valence lead individuals to process information markedly differently in terms of the content the individuals pay attention to and the depth of their thinking, which then greatly affect their judgments and decision-making. For example, individuals who experience fear tend to perceive a high level of uncertainty and uncontrollability in new situations, and this makes them perceive greater risks in these situations. In comparison, individuals who experience anger tend to perceive a low level of uncertainty and uncontrollability in new situations. Therefore, fearful individuals tend to select the option that is risk averse, whereas angry individuals tend to select the option that is risk seeking [8,9,10]. Furthermore, fear is a highly uncertain emotion. When individuals experience fear, they tend to adopt a systematic-processing mode to process information because they are unsure what will occur in subsequent situations. Therefore, carefully examining the information at hand allows them to avoid harm [8,24,25]. Conversely, anger differs significantly from fear because the former is an emotion with a high level of certainty and controllability. Therefore, when individuals experience anger, they tend to adopt a heuristic-processing mode to process information because they know what will occur in subsequent situations and do not worry about being harmed.

According to RISP, individuals’ affective responses will first positively elicit their perceived information insufficiency, which leads them to use a systematic-processing mode. Furthermore, RISP assumes that individuals’ affective responses have a direct impact on their adoption of a systematic-processing mode to process information [4,18]. COVID-19 is a mysterious disease with a rapid rate of transmission, and it can result in several sequelae if individuals contract this disease. Most people in Taiwan are highly threatened by this disease; thus, fear should be their commonly felt emotion [19]. Moreover, there is speculation regarding the origin of COVID-19 which claims that the virus was invented in a laboratory. Some people in Taiwan believe this rumor, and others do not. Thus, this study held that the emotion of anger would be felt by people in Taiwan. In line with this reasoning, the following hypotheses were developed:

**H3:**  *Respondents’ perceived fear is positively associated with their perceived COVID-19 information insufficiency.*

**H4:**  *Respondents’ perceived anger is positively associated with their perceived COVID-19 information insufficiency.*

As stated in RISP, negative emotions also exert a direct impact on individuals’ use of information-processing modes. Fear is a highly uncertain and uncontrollable emotion; thus, when individuals experience this emotion, they tend to adopt a systematic-processing mode to process information. In contrast, anger is a highly certain and controllable emotion; hence, when people experience anger, they are more likely to adopt a heuristic-processing mode to process information. Based on this reasoning, this study developed the following hypotheses:

**H5:**  *Respondents’ perceived fear is positively associated with their use of a systematic-processing mode to process information.*

**H6:**  *Respondents’ perceived anger is positively associated with their use of a heuristic-processing mode to process information.*

### 2.2. RISP, Information Subjective Norms, and Behaviors

The concept of ISNs came from the theory of planned behavior and was adopted by the RISP model [15]. The RISP model posits that individuals’ information need will be greatly influenced by those people who are important to them and who think they should have sufficient knowledge about a risk. The RISP model originally suggested that ISNs first activated individuals’ information insufficiency, which, in turn, affected their information-seeking behavior. However, later studies have demonstrated that ISNs not only activate people’s information insufficiency about a risk but also directly affect their information-seeking behavior [4,18,20,28]. For example, Hwang and Jeong (2020) investigated toxic chemicals in consumer products in South Korea and discovered that, as respondents reported higher levels of information norms, they were more likely to use a systematic-processing mode to process information [28]. The meta-analysis conducted by Yang et al. (2014) found that, when respondents perceived a lower degree of ISNs, they were more likely to adopt a heuristic-processing mode to process information [4].

Scholars define cultures as shared patterns of behaviors and cognitive concepts that greatly shape individuals’ perceptions, thinking, and behaviors in their social environments [29]. The most-studied cross-cultural dimensions are individualism and collectivism. Individuals in collectivistic cultures tend to define themselves based on social embeddedness and interdependence with others within their in-groups. Therefore, individuals in collectivistic cultures prioritize the needs, norms, and goals of their collectives, while people in individualistic cultures prioritize personal goals and preferences [29]. Empirical studies have shown that collectivism increases individuals’ inclination to behave in ways that further societal good rather than their personal interests [30]. For example, a study on 98 countries discovered that collectivism was negatively associated with confirmed cases of COVID-19 because individuals in collectivistic cultures were more willing to follow social norms related to taking preventive measures [31]. Following this reasoning, this study expected that ISNs would play a more significant role than other variables of the RISP model in collectivistic cultures such as Taiwan than in other cultures.

**H7:**  *Respondents’ ISNs are positively associated with their perceived COVID-19 information insufficiency.*

**H8:**  *Respondents’ ISNs are positively related to their use of systematic-processing modes of COVID-19 information.*

**H9:**  *Respondents’ ISNs are negatively related to their use of heuristic-processing modes of COVID-19 information.*

The RISP model is useful in predicting individuals’ information-seeking and -processing behavior. However, during the outbreak of COVID-19, a more important issue has been clarifying whether individuals’ active information seeking and processing allow them to form positive attitudes and behaviors toward COVID-19 prevention. Therefore, this study incorporated behaviors, which are the key variables in the HSM [16], into the RISP model to understand the consequences of information-seeking and -processing behaviors [1,32].

According to the HSM, when a systematic mode is employed, individuals rely on cognitive resources to process information; thus, they make a cognitive effort to thoroughly evaluate the information available, including the logic and evidence of a message, to form their judgments and decisions. Therefore, the attitudes and behaviors derived from using a systematic-processing mode are more stable and better able to resist counterarguments. In comparison, when a heuristic-processing mode is engaged, individuals rely less on cognitive resources to process information. Instead, they adopt a simple way to process information, usually using a shortcut to make judgments and decisions. Hence, the attitudes and behaviors resulting from using a heuristic mode are unstable and easy to change [4,16]. When COVID-19 peaked in Taiwan, people were situated in a context with high uncertainty; thus, they actively searched for relevant information about how to protect themselves. In addition, misinformation has been rife during this pandemic. With so much information available, it has been crucial for individuals to differentiate not only the true from the false but also the useful from the trivial to know how to help prevent the spread of COVID-19. Therefore, this study judged that the use of a systematic-processing mode allows individuals to critically analyze these pieces of information and identify useful and true information so that positive attitudes and behavior toward COVID-19 prevention can be established. However, when individuals use a heuristic-processing mode, which is a haphazard way to process information, they may not be able to handle all the information at hand and might thus experience a boomerang effect on their attitudes and behavior toward COVID-19 prevention. Following this reasoning, this study developed the following hypotheses.

**H10:**  *The use of a systematic-processing mode is positively associated with respondents’ information-seeking behavior toward COVID-19 prevention.*

**H11:**  *The use of a heuristic-processing mode is negatively associated with respondents’ information-seeking behavior toward COVID-19 prevention.*

## 3. Research Methodology: An Online Survey

### 3.1. The Online Survey

To collect a representative sample, the researchers hired a professional polling company, ETtoday, which has an online panel of 8.8 million members, to administer an online survey. Stratified random sampling was employed by using gender, age, education, personal income, and residential areas as strata to select participants from among the 8.8 million members. This study established two criteria for ETtoday to select potential participants: (1) they must be Taiwanese adults who are 20 years old or above and (2) the sample profile must be congruent with the characteristics of Taiwan’s online population. A recent study (TWNIC, 2020) showed that the internet penetration rate in Taiwan is approximately 88% for the overall population and that the age group with the highest rate, 96.73%, is people between 20 and 54 years old [33]. This online survey was conducted between 1 April and 18 April 2021, and 1100 valid questionnaires were obtained.

The questionnaire contained six parts (see Table 1). The first part contained four items to measure severity and susceptibility. The second part contained eight items to measure information-processing modes. Of these eight items, four items were for a systematic mode, and the remaining four items were for a heuristic mode. The third part contained six items, and each emotion was measured by three items. The fourth part contained four items to measure informational subjective norms. These items asked respondents the degree to which people important to them, including family members, friends, and relatives, expected them to seek COVID-19 information. The fifth part contained five items used to measure information-seeking behavior regarding COVID-19 prevention. These five items measured whether individuals paid attention to (1) the daily number of confirmed cases of COVID-19 in Taiwan, (2) the daily number of confirmed cases of COVID-19 in the world, (3) information regarding how to adopt self-protective measures against COVID-19, (4) information to help them understand the impact of COVID-19 on Taiwan, and (5) information to help them understand the impact of COVID-19 across the world.

The sixth part had two items. The first item asked the respondents to score their knowledge of COVID-19 from 0 to 100. The second item asked the respondents how much higher they would like their score to be (from 0 to 100) regarding how to take protective measures against COVID-19. Before the online survey was conducted, this questionnaire was sent to 20 individuals for a pretest. The results of the pretest showed that most subjects considered the last two items to be similar and suggested deleting one of them. Therefore, this study rephrased the second item to read, “If your perceived adequate knowledge of COVID-19 is 100, then how much higher score would you need to reach what you consider adequate knowledge?” This study deemed that this statement was similar to the definition of perceived information insufficiency by Griffin and Yang [4,18]. Therefore, the second item was used to measure individuals’ perceived COVID-19 information insufficiency.

The data-collection procedure for this study was approved by the university’s Research Ethics Committee for Human Subject Protection before data were collected, and the approval number is NCTU-REC-109-122 W.

### 3.2. Factor Analysis on Information Processing and Behaviors

This study adopted principal components and varimax rotation methods from the SPSS package to conduct two exploratory factor analyses (EFAs). The first EFA was conducted on the responses to the items on information-processing modes, and two factors were extracted. The first factor, a systematic-processing mode, contained four items that were concerned with how to carefully review and examine COVID-19 relevant information. The second factor, a heuristic-processing mode, contained four items related to using a superficial manner to process COVID-19 relevant information. The results of reliability analyses, summarized in Table 2, indicated that the Cronbach’s alphas for the two factors were 0.883 and 0.788, respectively. The second EFA was conducted on the responses to the items related to information-seeking behavior. One factor was extracted, and the Cronbach’s alpha for the reliability analysis was 0.926, thus indicating a high level of internal consistency.

### 3.3. Sample Profile

Among the sample, 562 (51.1%) respondents were males. Regarding education, 1.3% had obtained a junior-high-school education, 31.8% had obtained a senior-high-school education, 48.2% had obtained a college education, and the remaining 18.8% had obtained a graduate education. Regarding age, 19.4% of the respondents were between 20 and 29 years old, 21.9% were between 30 and 39 years old, 22.2% were between 40 and 49 years old, 19.7% were between 50 and 59 years old, and the remaining 16.8% were 60 or above. This sample profile was similar to that of Taiwan’s online population but differed from the general population in terms of the following: individuals older than 59 years and those who received a junior-high-school education were less well represented [37] .

## 4. Research Findings

**Figure 1:**
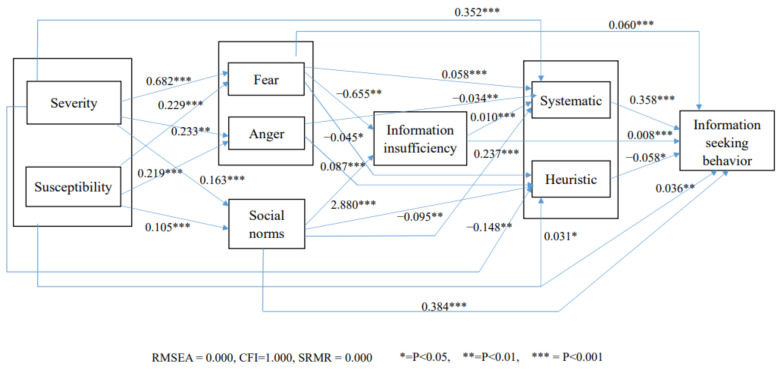
Path for information-seeking behavior.

The researchers conducted an SEM (structural equation modeling) by using the statistical software Mplus 7.4. [38] to examine the hypothesized relationships among the variables. Using the criteria suggested by statisticians—RMSEA (<0.08), SRMR (<0.08), and CFI (>0.95)—the results were RMSEA = 0.000, SRMR = 0.000, and CFI = 1.000, demonstrating a perfect model fit [39,40]. The data are summarized in Figure 1.

Figure 1 shows that the respondents’ perceived severity and susceptibility did not exert any significant effects on their perceived information insufficiency. Hence, H1 and H2 were not supported. For H3 and H4, the data in Figure 1 indicated that the respondents’ perceived anger did not exert any effects on their perceived information insufficiency, but their perceived fear exerted a significantly negative effect on information insufficiency. Therefore, H3 and H4 were not supported. Furthermore, Figure 1 demonstrates that the respondents’ perceived fear exerted a significantly positive impact on the use of a systematic-processing mode (γ = 0.058 ***) and a significantly negative impact on the use of a heuristic-processing mode (γ = −0.045 *). Conversely, perceived anger exerted a significantly positive impact on the use of a heuristic-processing mode (γ = 0.087 ***) and a significantly negative impact on the use of a systematic-processing mode (γ = −0.034 **). Thus, H5 and H6 were supported. For ISNs, Figure 1 indicates that ISNs were positively associated with respondents’ information insufficiency (γ = 2.880 ***) and their use of a systematic-processing mode for information processing (γ = 0.237 ***) but negatively associated with their use of a heuristic-processing mode for information processing (γ = −0.095 **). Hence, H7, H8, and H9 were supported. For information-seeking behavior, this study found that the use of a systematic-processing mode was positively associated with this variable, but the use of a heuristic-processing mode was negatively associated with it. Therefore, H10 and H11 were supported.

Several additional findings are worth noting: (1) Respondents’ perceived severity exerted a positive effect on the use of a systematic-processing mode (γ = 0.352 ***) but a negative effect on the use of a heuristic-processing mode (γ = −0.148 **). Perceived susceptibility exerted a positive effect on the use of a heuristic-processing mode (γ = 0.031 *) and information-seeking behavior (γ = 0.036 **). Furthermore, both perceived severity (γ = 0.163 ***) and susceptibility (γ = 0.105 ***) exerted a positive effect on ISNs. (2) Fear exerted a positive effect on information-seeking behavior (γ = 0.060 ***). (3) Information insufficiency exerted a significantly positive effect on the use of a systematic-processing mode (γ = 0.010 ***) and information-seeking behavior (γ = 0.008 ***). (4) ISNs exerted a significantly positive effect on information-seeking behavior (γ = 0.384 ***).

## 5. Discussion

### 5.1. Risk Perception, Affective Response, and ISNs on Information Insufficiency

This study unexpectedly discovered that risk perception, including perceived severity and perceived susceptibility, did not exert any significant impacts on respondents’ perceived information insufficiency, and this is not congruent with the assumptions of the RISP model [4,5,23]. According to the RISP model, the most important variables in this model are risk perception and affective response. Risk perception is derived from risk characteristics, while affective response includes several specific emotions. In particular, risk perception is regarded by RISP as the most critical variable, and it exerts both a direct and an indirect impact on respondents’ perceived information insufficiency, which then affects their information-seeking and -processing behavior. This study not only found that risk perception did not exert any significant effects on information insufficiency but also discovered that affective responses, including fear and anger, were not positively associated with respondents’ information insufficiency. Conversely, fear was shown to have a negative effect on respondents’ information insufficiency, indicating that, when respondents were more fearful, they perceived a lower degree of need for information. These findings indicated that risk perception did not have any impacts either directly or indirectly on respondents’ information insufficiency, and this is not congruent with the predictions of RISP.

However, this study found that risk perception exerted a powerful impact on respondents’ ISNs, and this, in turn, significantly and positively affected their information insufficiency. That is, both respondents’ perceived severity and perceived susceptibility exerted a significantly positive impact on ISNs, and this then positively activated their perceived information insufficiency. A plausible interpretation for these unexpected findings is that the respondents of this study were so concerned about ISNs that the impact of ISNs overshadowed that of the other variables in RISP. More specifically, Taiwan has a collectivistic culture, and the people cared very much about what other people were thinking about them during the outbreak of COVID-19. Therefore, this study did not observe any significant impacts arising from risk perception and affective response on respondents’ information insufficiency. Instead, information insufficiency was significantly activated by respondents’ ISNs. This study further examined the R^2^ explained by each set of the variables on the dependent variable—information-seeking behavior—to verify this interpretation. The results indicated that the five sets of variables—risk perception, affective response, ISNs, information insufficiency, and information processing—had a summed adjusted R^2^ of 0.444, indicating that the five sets of variables accounted for approximately 44% of the variance in information-seeking behavior. ISNs’ R^2^ was 0.221, showing that ISNs explained almost half of the total variance for information-seeking behavior. Hence, these findings demonstrated that ISNs in collectivistic cultures have become so powerful during the COVID-19 pandemic that this variable has strongly activated respondents’ need for information on this pandemic, which is consistent with the findings of several past studies that were also conducted in collectivistic cultures. For example, Li and Zheng (2020) found that respondents’ ISNs exerted a significant and positive impact on their information insufficiency during the COVID-19 outbreak in China, and this subsequently positively affected their online information seeking. Similarly, Lu et al. (2021) recruited US-dwelling Chinese people as respondents and examined how they relied on different evidence types for information seeking during the outbreak of COVID-19 [30]. Their study found that ISNs exerted a significant impact on respondents’ perceived information insufficiency, which then positively affected their information-seeking behavior. Another example is the study by Hwang and Jeong (2020), which discovered that ISNs were positively associated with respondents’ information-seeking behavior regarding toxic chemicals in consumer products in South Korea [28].

In addition, ISNs were found to be positively associated with the use of a systematic-processing mode but negatively associated with the use of a heuristic-processing mode for processing COVID-19 information; this finding is congruent with the predictions of H8 and H9. This study also discovered that ISNs had a direct impact on respondents’ information-seeking behavior.

In summary, this study found that ISNs played a significant role in respondents’ information-seeking and -processing behavior, not only activating people’s strong need for information and leading respondents to adopt an active approach in information processing but also directly affecting their information-seeking behavior. Future studies should further investigate the relationship between ISNs and collectivism/individualism to better understand how cultural orientations influence individuals’ information-seeking and -processing behavior during a risk event. In particular, individuals vary in their degrees of collectivism even within the same collectivistic cultures, which may affect the role of ISNs in the RISP model.

### 5.2. Effects of the Five Sets of Variables on Behavior

As predicted by H5 and H6, this study discovered that fear is a highly uncertain and highly uncontrollable emotion; thus, the respondents who experienced this emotion were found to have a high probability of using a systematic-processing mode to process information. In comparison, anger is a highly certain and highly controllable emotion; hence, the respondents who experienced this emotion were more likely to adopt a heuristic-processing mode. These findings are congruent with the assumptions of the ATF that emotions sharing the same valence—negative emotion—lead individuals to assess a risk in completely different manners, which, in turn, yield markedly different judgments and decisions [8]. Similarly, Yang et al.’s study (2019) showed that anger exerted no effects on respondents’ adoption of a systematic-processing mode in either the election or climate change samples [7].

Moreover, respondents’ fear was found to exert a negative impact on their information insufficiency, but information insufficiency exerted both a direct and an indirect impact on information-seeking behavior, indicating that too much fear might produce a boomerang effect on respondents’ active information-seeking behavior. However, the data in Figure 1 show that fear exerted a positive and direct effect on information-seeking behavior, a positive effect on the use of a systematic-processing mode, and a negative effect on the use of a heuristic-processing mode, all of which were conducive to respondents’ active information-seeking behavior. Therefore, fear, in general, was helpful in activating respondents’ effortful information-seeking behavior toward COVID-19 prevention. Conversely, anger was discovered to exert a negative effect on the use of a systematic-processing mode and a positive effect on the use of a heuristic-processing mode, both of which were detrimental for respondents’ effortful information-seeking behavior. Therefore, during the outbreak of a risk, eliciting too much anger from the public may not be effective because experiencing too much anger does not allow individuals to actively search for relevant information to avoid harm.

As predicted by H10 and H11, this study discovered that respondents’ use of a systematic-processing mode was positively associated with information-seeking behavior regarding COVID-19 prevention. In contrast, the use of a heuristic-processing mode was negatively associated with information-seeking behavior. These findings are congruent with the assumptions of the HSM, indicating that when respondents adopted a systematic-processing mode, more cognitive resources were utilized to evaluate the logic and evidence of the information; thus, they were able to see the detrimental impact of contracting COVID-19 and further identify those effective measures to protect themselves. Therefore, a positive relationship between the use of a systematic-processing mode and behaviors regarding COVID-19 prevention was discovered by this study. Instead, when a heuristic-processing mode was adopted, respondents utilized fewer cognitive resources to process information so that they were unable to understand the negative consequences of contracting the disease. As a consequence, the use of a heuristic-processing mode was negatively associated with respondents’ information-seeking behavior regarding COVID-19 prevention. These findings are also consistent with those of prior studies, demonstrating that using a systematic-processing mode was positively associated with attitudes and behaviors toward the prevention of risks [7,17,35]. For example, Griffin et al. (2002) examined three risks—a fish hazard, a tap water hazard, and an ecological risk to a lake—and found that the use of a systematic-processing mode was positively associated with the subjects’ number of strongly held beliefs, evaluation strength, and cognitive structure strength, while the use of a heuristic-processing mode was negatively correlated with the three outcome variables [35]. In addition, Li and Zheng (2020) discovered that, as respondents were more active in seeking information about COVID-19 than about other topics, they had stronger attitudes and higher degrees of intentions to adopt preventive measures to protect themselves [1]. Although Li and Zheng (2020) did not use the variable of information-processing modes, more active information seeking was indeed related to the use of systematic-processing modes [1].

Although risk perception, including perceived severity and perceived susceptibility, was not associated with respondents’ information insufficiency, this study still found that risk perception played a significant role in respondents’ information-seeking and -processing behavior because risk perception exerted an indirect impact on information-seeking behavior through ISNs. In particular, perceived severity was found to play a more important role than perceived susceptibility in information-seeking behavior because, in addition to significantly impacting ISNs, perceived severity was positively associated with the use of a systematic-processing mode but negatively associated with the use of a heuristic-processing mode. Perceived susceptibility was found to exert a positive effect on the use of a heuristic-processing mode and have a direct effect on respondents’ information-seeking behavior. These findings are congruent with the assumption of RISP, showing that risk perception is the most critical variable in the RISP model because it not only activates individuals’ need for information seeking but also determines how individuals handle the obtained information [4,5,24].

Furthermore, this study discovered that respondents’ perceived information insufficiency played a limited role in RISP because, among the five sets of variables, information insufficiency had the least amount of explanatory power, accounting for only 1.5% of the variance in information-seeking behavior. This finding is congruent with Yang et al.’s meta-analysis (2014), showing that current knowledge, rather than information insufficiency, consistently predicts information-seeking and -processing behaviors [4]. Yang et al. (2014) suggested that, for familiar risks, information insufficiency was capable of predicting outcome variables [4]. However, for unfamiliar risks, it was difficult for individuals to estimate how much more information was needed for them to adequately manage a potential risk. As indicated in Zhou’s study (2021), COVID-19 was unfamiliar; thus, his study used perceived information needs instead of perceived information insufficiency to predict information seeking and found that information needs were indeed a powerful predictor of information-seeking behaviors [23].

## 6. Conclusions

This study adopted the RISP model as a theoretical framework to investigate the factors that prompted individuals’ information-seeking and -processing behaviors during the COVID-19 pandemic. The data analysis yielded four conclusions: (1) ISNs were the most powerful predictor among the five sets of variables because ISNs not only activated respondents’ need for information and led them to adopt an active approach in information processing but also exerted a direct impact on respondents’ information-seeking behavior; (2) fear was conducive to activating respondents’ active information-seeking behavior, while anger had a boomerang effect on respondents’ effortful information-seeking behavior; (3) perceived severity played a more important role than perceived susceptibility in respondents’ information seeking and processing during the studied period of the COVID-19 pandemic; and (4) information insufficiency played a limited role in RISP during the studied period of the COVID-19 pandemic partly because it was an unfamiliar issue for people in Taiwan.

## Figures and Tables

**Table 1:** Items for major variables.

Variable	Number of Items	Reference
Severity and susceptibility	4	Witte (1994) [34]
Information-processing modes	8	Griffin et al. (2002) [35]
Emotions	6	Kahlor et al. (2006) [36]
Informational subjective norms	4	Hwang and Jeong (2020) [28] Li and Zheng (2022) [1]
Information-seeking behavior	5	
Information insufficiency	2	Yang & Griffin [4,18]

All variables were measured by using a Likert scale of 1–7.

**Table 2:** Results of factor analysis on information processing.

	Factor 1 Loading	Factor 2 Loading
Factor 1: A systematic-processing mode		
Carefully thought about COVID-19	0.875	−0.140
To act on this matter, the more viewpoints I get the better	0.847	−0.134
Broader understanding after reading the news about COVID-19	0.800	−0.109
Carefully considered the perspectives of the news about COVID-19	0.891	−0.120
Factor 2: A heuristic-processing mode		
Did not spend much time thinking about COVID-19	−0.117	0.727
Do not need so much information on COVID-19	−0.145	0.838
Focus only on a few key points of the information about COVID-19	−0.081	0.808
To act on this issue, the advice of one expert is good enough for me	−0.114	0.720
Eigenvalue	3.541	1.894
Variation explained (%)	44.261	23.68
Cronbach’s alpha	0.788	0.883

## Data Availability

Please contact the corresponding author for the information of data availability.
